# Prostate Cancer Immunotherapy: Exploiting the HLA Class II Pathway in Vaccine Design

**DOI:** 10.4172/2155-9899.1000351

**Published:** 2015-08-26

**Authors:** Bently P Doonan, Azizul Haque

**Affiliations:** Department of Microbiology and Immunology, and Hollings Cancer Center, Medical University of South Carolina, 173 Ashley Avenue, Charleston, SC 29425, USA

**Keywords:** Prostate cancer, Tumor associated antigens, HLA class II proteins, Cysteinylation, Gamma-Interferon-inducible lysosomal thiol reductase, Immunotherapy

## Abstract

Prostate cancer is the second most diagnosed cancer in men and current treatment of advanced prostate cancer is ineffective. Immunotherapy has emerged as a promising treatment option for metastatic prostate cancer but its clinical application is still in the early stages of development. In order to treat metastatic prostate tumors, new directions must be taken to improve current immunotherapeutic strategies. These include the identification of effective tumor antigens (Ags), the induction of the HLA class II pathway for Ag processing and CD4^+^ T cell activation, and the ability of tumor cells to act like Ag presenting cells. In this review, we suggest a model for tumor Ag selection, epitope modification and self-processing for presentation by class II proteins as a means of restoring immune activation and tumor clearance. We also outline the importance of a Gamma-IFN-inducible Lysosomal Thiol reductase (GILT) in Ag and modified peptide processing by tumor cells, generation of functional epitopes for T cell recognition, and inclusion of immune checkpoint blockers in cancer immunotherapy. Taken together, this review provides a framework for the future development of novel cancer vaccines and the improvement of existing immunotherapeutics in prostate cancer.

## Introduction

Prostate cancer is the second most common diagnosed cancer in men with close to 200,000 new cases reported in the US annually [1,2]. Strategies employed for treatment include hormone therapy, surgery, radiation, and chemotherapy [2–4]. Though useful, these therapies only allow temporary relief and have minimal long-term impact on late-stage metastatic prostate cancer. This lack of effective treatment opens the door to new options like immunotherapy, and combination of chemotherapy and immunotherapy for the treatment of metastatic prostate tumors [5,6]. Recently, US Food and Drug Administration (FDA) approved a promising immunotherapeutic regimen for treating metastatic hormone-refractory prostate cancer, the dendritic cell therapy Provenge (Sipuleucel-T, Dendreon) [7–10]. Unfortunately, this treatment strategy has shown only minimal increases in survival outcomes limited to about 4 months, and has a hefty price tag associated with it that isn’t without critique [11–13]. Some of the issues associated with Provenge’s efficacy will be addressed in this review. Recently, a phase 3 clinical trial assessing ipilimumab efficacy in castration-resistant disease also showed no clear efficacy [14,15]. In order to more effectively treat prostate cancer with immunotherapy there are many factors that need to be addressed and improved before it becomes a viable option [16]. These factors include the identification of effective tumor associated antigen (TAA) that can activate both the innate and adaptive immune system resulting in a strong immune response and the development of immunological memory. Currently most if not all immunotherapeutics are designed to induce antigen (Ag)-specific cytotoxic CD8^+^ T cells (CTL). Less attention has been given to the activation of CD4^+^ T cells although these cells play a major role in initiating and maintaining CTL activity [17–19]. New immunotherapeutic approaches must also address the problems associated with Ag induced T cell tolerance, how to overcome cancer cells’ poor Ag presentation capability, and how to prevent or reverse the immune evasion mechanisms employed by prostate cancer cells.

First, an effective tumor derived Ag must be identified before a suitable immunotherapeutic treatment can be established. Fortunately, there are many good candidates for prostate tumor Ags; including prostate specific antigen (PSA), prostatic acid phosphatase (PAP), prostate specific membrane antigen (PSMA), telomerase, and survivin [20–31]. Each Ag will be discussed in this review with the main focus on PSMA and survivin. Our laboratory has previously shown cysteine containing Ags are susceptible to cysteinylation which may lead to Ag induced T cell tolerance [32], this topic will be discussed in more detail in this review.

One of the pitfalls of current immunotherapeutic strategies is that the therapies largely focused on the HLA class I pathway and CD8^+^ T cell recognition of tumor cells. While this pathway is of great importance as it is responsible for direct tumor killing through cytotoxic lymphocyte activity, it cannot sustain a long-term immune response and prolonged killing of tumors by itself, accounting for the sporadic results of class I cancer vaccine trials [33]. Thus, the HLA class II pathway should be considered in designing immunotherapy in order to have a complete and sustained antitumor response. While the importance of the HLA class II pathway has been well defined in autoimmune diseases [34], cancer immunotherapeutics designed to improve this system have been few and far between, with few exceptions [26,35]. We have recently shown that prostate cancer cells express HLA class II molecules that can be recognized by CD4^+^ T cells [36]. In this review, we propose a framework for including both class I and class II pathways in future prostate cancer immunotherapy through careful selection of tumor Ags, understanding of cysteinylation in epitope modification, and the presence of gamma-interferon-inducible lysosomal thiol reductase (GILT) in tumor Ag processing and presentation. We have previously shown that the induction of GILT into melanoma cells increases their ability to behave like professional antigen presenting cells (APCs) [37], and if prostate cancer cells could be likewise effectively turned into APC through the introduction of GILT, then the efficiency of immunotherapy would improve greatly. This information when put together may provide a good framework for the design of new generation immunotherapeutics against metastatic prostate cancer.

## Prostate Cancer Associated Ags

There are many identified potential prostate tumor associated antigens (TAA) that could be exploited for immunotherapy. Table 1 highlights a few important prostate TAAs that have received attention for use in prostate cancer immunotherapy [26,28,31,38,39]. PSA is one of the first discovered prostate tumor derived Ags, and has direct implications in the clinical setting as PSA levels are monitored as a means of staging cancer progression and testing therapy efficacy. However, PSA-based prostate staging and detection is not perfect as there are issues with false positive results [40,41]. Much research has been directed at manipulating PSA, including the prostate cancer vaccine PSA-TRICOM (Prostvac), which showed an improvement of overall survival in a phase II clinical trial [42,43], and a phase III randomized trial is underway. PAP also has clinical significance and has been used recently in Ag loaded APC immunotherapy clinical trials [44]. PAP is found in both secretory and cell-associated forms and is present in normal prostate tissue, and to some extent in other body tissue [24]. Cellular PAP is down-regulated in carcinomas while the serum concentration of PAP increases dramatically and until the advent of PSA as a better marker, PAP was used as a serum marker for prostate cancer [39]. It is thought that PAP has growth suppressing factors that cancer cells down regulate to improve division increasing the amount of shed PAP in serum through malignant disruption of prostate epithelium [45]. PAP is also used as a fusion protein coupled with GM-CSF in the dendritic cell (DC) therapy Provenge [7]. Though results of Provenge clinical trials display a 4 month survival advantage over chemotherapy alone, there are some issues with its design and application. The procedure requires isolation of patient DC for co-stimulation ex vivo with the PAP-GM-CSF protein where GM-CSF targets the GM-CSF-receptor expressing DC allowing for internalization and processing of PAP for display when reinjected into the patient [42]. Upon reinjection, this DC-presented PAP peptide can stimulate T cells mounting an antitumor immune response. However, this response is not seen in every patient and the durability of the immune response is also questionable given the low time to progression (TTP) rates observed (Provenge TTP 11months, control arm 10 months) [42]. Possibly, the selection of PAP as the target Ag may limit this technique’s efficacy and through selection of more immunogenic peptides, or through the application of multiple prostate specific peptides, a more substantial response might be observed. Prostate Stem Cell Antigen (PSCA) has also been investigated as TAA or biomarker for diagnosis and therapy of malignant prostate tumors [46]. PSCA is a cell-surface glycosylphosphatidylinositol-anchored protein expressed in prostate as well as other malignancies. Studies suggest that PSCA is highly expressed in majority of human prostate cancer, and could be associated with transformation of prostate cells and tumorigenesis [47,48]. Thus, PSCA is thought to be an important target in advanced prostate cancer. Recently, PSCA has shown clinical potential in immunotherapy because this TAA is presented by dendritic cells to induce strong antitumor immunity. PSCA antibody-based immunotherapy has also shown some promise in mouse model of prostate cancer [49]. While HLA class I-restricted PSCA epitopes have been identified and shown to enhance CD8^+^ T cell responses [50], the nature of HLA class II-restricted PSCA epitopes in eliciting CD4^+^ T cell responses remain unclear.

PSMA is produced by normal prostate epithelial cells at low levels [20], but is overexpressed in metastatic prostate carcinomas. PSMA is a ubiquitous cancer Ag expressed in many different cancers not just prostate; such as breast, ovary, kidney, and lung making it a good target for immunotherapy as it has broad specificity [26]. The immunodominant epitope of PSMA, PSMA_459_, has also been isolated and shown to induce both CD4^+^ and CD8^+^ responses [26]. These factors make PSMA a good target for prostate cancer immunotherapy. Work performed by Schroers and Shen et al. identified the class II-restricted dominant epitope of PSMA and it represents an ideal method for Ag selection [26]. In their study, TEPITOPE software was used to predict potential promiscuous HLA-DR-binding regions [26]. From this, six sequences with the PSMA amino acid structure were found to have promiscuous HLA-DR binding: PSMA_17_, PSMA_100_, PSMA_206_, PSMA_459_, PSMA_576_, and PSMA_730_ [26]. These peptides were then synthesized and analyzed for their ability to elicit a CD4^+^ T cell response from Peripheral Blood Mononuclear Cells, (PBMCs) donated by varying DR typed healthy individuals, including DR1, DR4, DR7, and DR11 [26]. All six peptides were found to be reactive but under statistical scrutiny only four of the six peptides, PSMA_206_, PSMA_459_, PSMA_576_, and PSMA_730_, were selected as potential CD4^+^ T cell epitopes [26]. From this study, the group found that only PSMA_459_ represented a naturally processed HLA class II-restricted epitope defining it as the immunodominant epitope [26]. This strategy of Ag/ epitope discovery could be exploited for other prostate tumor Ags that may have a better defined role in tumor survival such as telomerase and survivin.

Telomerase is a transcriptase responsible for telomere length that is found in about 90% of all cancers [30]. Telomerase has been shown to be vital to the immortalization of cancer cells as it allows tumors to proliferate without undergoing apoptosis or cell degradation [51]. When present in tumor cells the telomerase generated protein TERT is processed and presented through the HLA class I pathway and is capable of stimulating CD8^+^ T cells [38]. The telomerase vaccine GV-1001 has seen the most widespread testing of a telomerase based immunotherapy, displaying a preferential toxicity profile in stage I/II clinical trials in pancreatic cancer patients [51]. Unfortunately, results have only shown a 50–80% response rate, with little clinical benefits [51]. However, these results are preliminary and given TERT’s almost exclusive expression in tumor cells make telomerase an ideal target for prostate cancer therapy but more work is needed, as current investigation is in its infancy [38]. Like telomerase, survivin is a vital element to cancer cell survival and a good target for prostate cancer immunotherapy. Survivin is an antiapoptotic protein overexpressed by many different cancers, including breast, brain, melanomas, many leukemias and lymphomas, colorectal, and prostate [52,53]. Survivin is also extremely low or undetectable in normal healthy tissues and is mainly found in thymocytes, bone marrow derived hematopoietic cells, basal colonic epithelial cells, and activated endothelial cells [54]. Survivin acts by blocking mitochondrial-dependent caspase 9 activity, preventing programmed cell death [27,54].

Through inhibition of apoptosis and increased cell division, survivin also plays an important role in the formation of a tumor’s mass and ultimately advanced staging of prostate cancer with a poor prognosis [54]. However, since survivin seems to play such an important role in cell division and appears to be necessary to tumor cell survival, if it can be exploited then immunotherapy aimed at survivin would be effective at any stage of prostate cancer and in many different types of cancer [55]. Unlike PSMA, the immunodominant epitope of survivin has yet to be defined. Much work has been done to find immunodominant epitope(s) in cancer and there are many potential candidates [27]. Survivin also possesses HLA class I and II-restricted epitopes, and have been show to induce robust CD4^+^ T-cell responses in the majority of vaccinated cancer patients [56,57]. In studies carried out by Wang et al. [57], given survivin’s short length (142 amino acids), a set of 27 overlapping peptides encompassing the entire sequence was synthesized and analyzed through binding assays specific to HLA-DR and HLA-DP4 molecules [27]. In order to improve epitope binding, potential each peptide overlap was designed to contain one aliphatic or aromatic residue in one of its first 5 positions as DR and DP4 binding specificity calls for [27]. The study then rated each peptides binding capacity for specific DR and DP4 alleles, illustrating that some peptides were allele specific and others had broad specificity across DR alleles [27]. Next, alleles with broad specificity were assessed for their ability to elicit a T cell response in healthy donor PBMCs. This study showed that specific class II-restricted survivin epitopes were able to generate a CD4^+^ T cell response, but an immunodominant epitope was not identified as the study focused more on immune prevalence than dominance [27]. Instead, a list of potential peptides (survivin peptides 17–31, 90–104, 96–110, 128–142) to be included in cancer vaccine studies was given based on their immune prevalence and ability to elicit both a CD4^+^ and CD8^+^ response [27]. Whether or not these specific peptides prove to be the immunodominant epitope is less relevant than the fact that this study identified potential class II-restricted epitopes that could be exploited in prostate cancer immunotherapy. This initial work on immune regulation could serve as the foundation for potential prostate cancer vaccine trials that look to elicit a complete HLA class I and HLA class II immune response.

## Cysteine Reduction of Tumor Ags and Peptides

In order to be fully effective, some antigenic peptides must be internalized and processed by APCs. These peptides seem to have a common feature in that they usually contain one or more cysteine residues [58]. Cysteines in antigenic proteins or peptides are susceptible to cysteinylation reactions which occur spontaneously when the cysteine interacts with cystine in body fluid forming a cystine dimer. These reactions can take place outside of the cell prior to internalization into endocytic compartments. Cysteinylation is an oxidation reaction that changes the conformation of the protein/ peptide through the formation of disulfide bonds which expose new binding domains of HLA class II Ags to APCs. This phenomenon has been investigated previously by our group, showing that the cysteine-containing protein Ags/peptides alter T cell response to class II epitopes [59].

**Figure 1:**
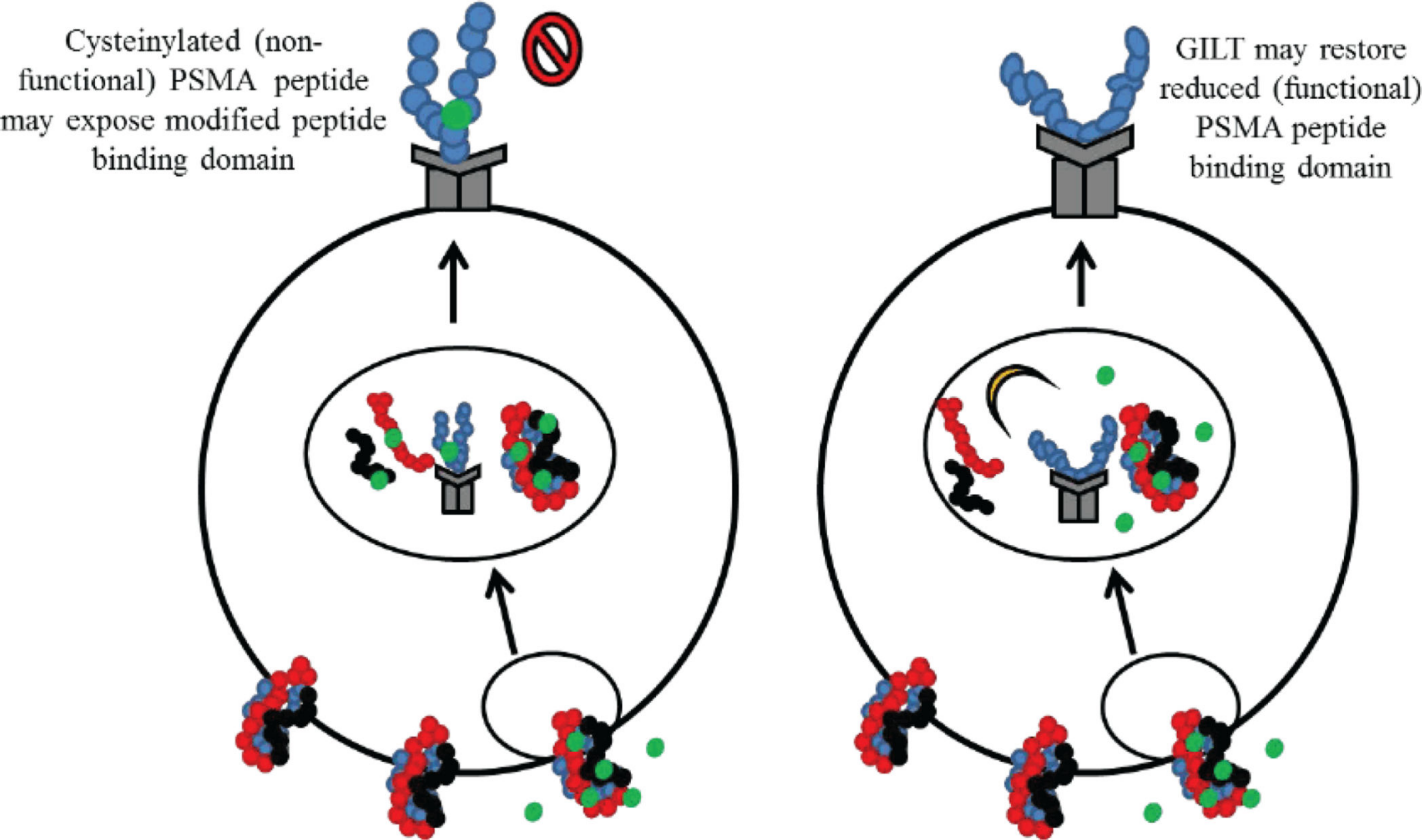
A schematic diagram showing possible cysteinylation of PSMA protein and reductive cleavage of PSMA by GILT in prostate cancer cells. The prostate specific membrane protein PSMA or its peptides can be oxidized in the presence of cystine in bodily fluid and that Ags/peptides remain non-functional in the absence of reductive processing by GILT. The results of which could be a differential selection and display of HLA class II-peptide complexes on the surface of prostate cancer cells, lowering CD4^+^ T cell recognition. The introduction of GILT in prostate cancer cells may lead to reduction and processing of the cysteinylated Ags/peptides, restoring functional PSMA presentation and improved CD4^+^ T cell recognition of prostate tumors.

Cancer cells load their surface HLA molecules with cysteinylated peptides as ligands as a way to avoid T cell recognition inducing tolerance. Once this occurs, the peptide is effectively muted and once loaded onto the HLA molecules, cysteinylation is irreversible. Our laboratory has documented that cancer cells display deficient Ag processing capability [60], potentially leading to their poor Ag presentation and minimal T cell response to tumor derived Ags that contain cysteine residues [61]. PSMA_459_ is one such peptide that contains a central cysteine residue and could be cysteinylated by cystine in body fluid. The presence of a reductase, GILT, in cancer cells may reduce the cysteinylated PSMA peptide(s) back to its functional form, leading to enhanced immune recognition and tumor clearance (Figure 1). We have recently shown that prostate cancer cell lines as well as primary prostate tumors express detectable levels of class II molecules. As shown in Figure 1, prostate cancer cells that naturally express PSMA may interact with free floating cystine in body fluid as PSMA is a membrane bound protein. Following internalization of this peptide and endosomal processing, bound cysteine residues will fail to be reduced in the absence of the thiol-reductase GILT. This could result in the presentation of cysteinylated peptides that are unable to stimulate CD4^+^ T cells aiding in immune avoidance. The cysteinylated peptide that binds to HLA class II molecules with a higher affinity is nearly impossible to modify or reverse. However, as seen in other tumors, the introduction of GILT in prostate cancer cells may overcome inefficient processing of cysteinylated peptide(s) by reducing peptides in the acidic endosomal and lysosomal compartments. A functional class II-PSMA complex may then be formed on the cell surface to elicit a better CD4^+^ T cell response. This finding suggests that a novel method of tumor Ag derivation could be achieved through the introduction of GILT in prostate cancer cell lines or in primary prostate tumors. These cells could then process prostate tumors Ags either endogenously during culture or through co-incubation with target Ags. Peptides derived from this Ag would differ from the naturally occurring tumor Ags and could be reintroduced into the patient following cancer cell irradiation to prevent further growth or division as a means of stimulating an immune response. This whole-cell cancer vaccine approach has been utilized in the past, most notably in the form of the GVAX vaccine. In this approach, transfected prostate cancer cell lines (PC-3 and LNCaP) have been used to express high levels of GM-CSF that could non-specifically boost DC antitumor activity when reintroduced into the patient [42]. Following promising phase I/II clinical trials, GVAX was tested in a large phase III clinical trial in combination with chemotherapy against conventional chemotherapy alone [42]. Unfortunately, GVAX clinical trials were halted due to an increased number of deaths observed in the GVAX arm versus the chemotherapy control arm [42,62]. To avoid these issues with whole-cell cancer vaccines, an alternative use of GILT-transfected cells could be explored with identification of novel tumor Ags derived from GILT-expressing cells that could be used in protein/peptide vaccine strategies.

## HLA Class II Processing in Prostate Cancer Cells

HLA class II protein expression in prostate cancer cells has been an issue of contention in recent years, adding to the limited study of class II vaccine strategies for prostate cancer. Previous study by Nanda et al. showed variable HLA class I and class II expression in human and mouse prostate tumors as well as localized to the tumor microenvironment [63]. Their study also showed strong correlation between HLA class I and class II expression rates between transgenic andenocarcinoma of the mouse prostate (TRAMP) tumors and human prostate tumors [63]. They concluded that while HLA class I is expressed on tumors, HLA class II is only present in hematopoietic lineage cells in the microenvironment [63]. These results could be due to differences in altered expression of the HLA class II master regulatory gene, class II transactivator (CIITA), which also governs HLA-DM and invariant chain (Ii) expression, and are also altered in prostate cancer cells [36]. Mishra et al. recently found aberrant CIITA gene expression in prostate cancer cell lines related to increased methylation rates, potentially leading to the observed changes in HLA class II protein expression [64]. However, our own study reported stable class II protein expression in human prostate cancer cell lines capable of directly activating T cells, suggesting that the HLA class II pathway should be taken into consideration for future prostate cancer vaccine design [36].

The class II molecule itself is an αβ heterodimer synthesized in the endoplasmic reticulum and expressed on the surface of professional APCs and to some degree on cancer cells [63]. The HLA complex is transported from the endoplasmic reticulum through the trans-Golgi apparatus to the acidic endosomal and lysosomal compartments where class II is processed and loaded with antigenic peptides. These acidic compartments are home to a variety of cathepsins and reductases that further refine antigenic peptides into smaller ligands that are now capable of HLA class II loading and presentation by APCs to CD4^+^ T cells [37,65,66]. This is also the site where reduction of cysteinylated peptides may occur and where GILT’s influence improves Ag processing and epitope generation. Once the class II-peptide complex is formed, it is transported to the cell surface for presentation to CD4^+^ T cells. Our laboratory has recently shown that class II protein expression is significantly increased in prostate cancer cells if they are cultured in hormone enriched media [36]. Thus, HLA class II-restricted prostate tumor Ags must be selected for therapy that can also induce a helper response not just a HLA class I response. Tumor cells themselves also need to be manipulated in order to improve their HLA class II processing and direct presentation ability. Cancer cells cannot functionally process and present antigenic peptides in the same way that professional APCs do, this is thought to aid in the immune evasion of cancer cells. The majority of cancer cells do not express or express very low levels of GILT, altering their Ag processing capability [36]. Cancer cells also lack costimulatory molecules like CD80 and CD86 that are vital to improving the fit of T cell receptors with the class II-ligand complex [17]. The absence of these factors inhibits the effect of CD4^+^ T cells and limits the efficacy of immunotherapy [67].

**Figure 2:**
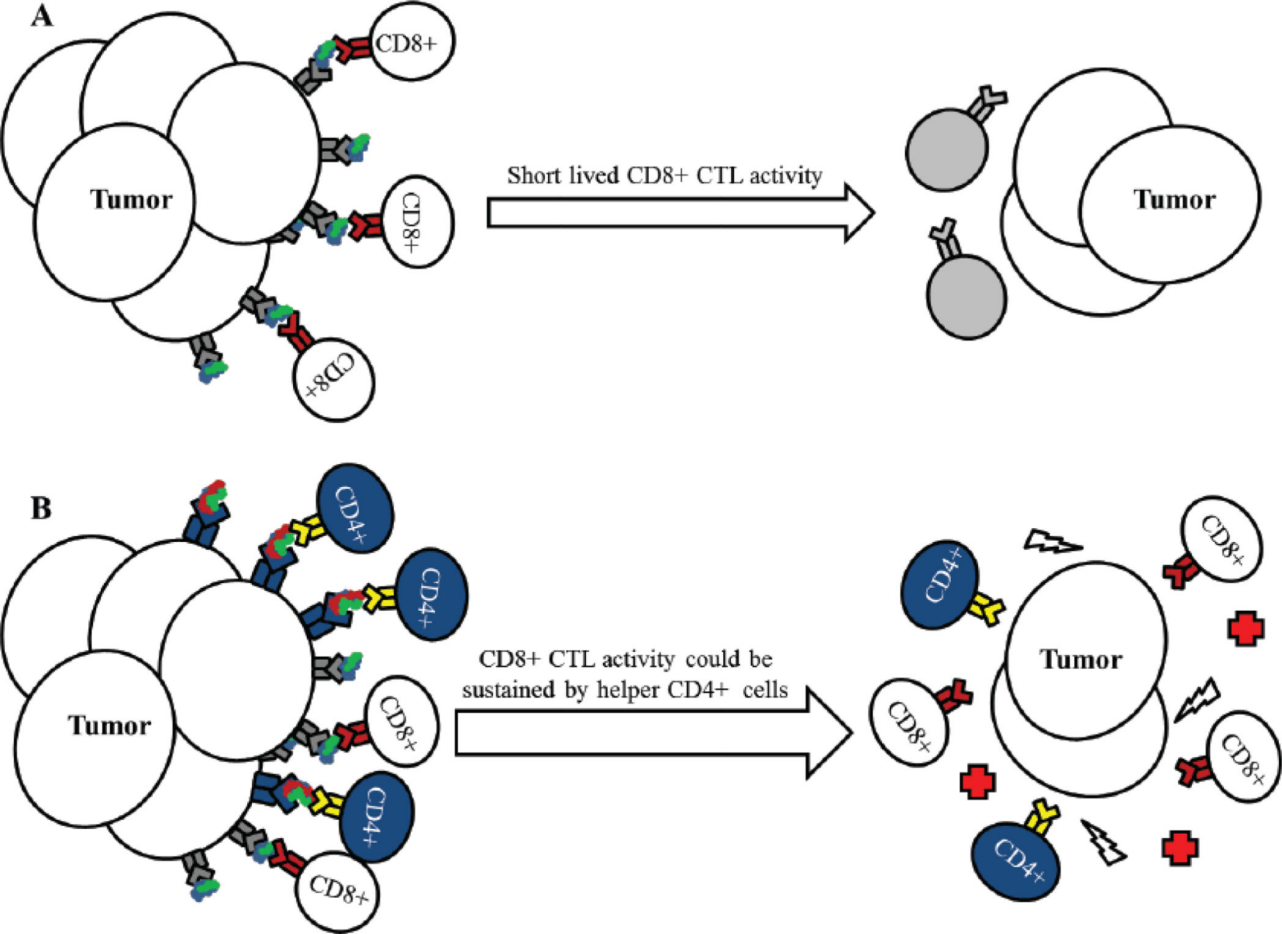
HLA class II-restricted helper CD4^+^ T cell responses can prolong CD8^+^ CTL activity. (A) CD8^+^ T cells recognize HLA class I-peptide complexes on tumor cells and directly kill cancer cells, reducing tumor burden. However, this reaction is not sustained for very long and the remaining tumor mass may continue to divide and progress. (B) Tumor cells that express HLA class II proteins or are induced to express class II proteins, can stimulate CD4^+^ T cells resulting in prolonged CD8^+^ CTL response and sustained tumor killing through the release of cytokines and immune stimulatory molecules.

HLA class II-restricted CD4^+^ T cells play a vital role in providing help to B cells in immunoglobulin switching and affinity maturation, activation and prolonged stimulation of CD8^+^ cytotoxic lymphocytes, and in expanding and persisting memory cells [68]. CD4^+^ T cells can also directly kill pathogens through release of cytokines and through cell-mediated cytotoxicity [69]. The most important characteristic of CD4^+^ T cells in immunotherapy being their ability to sustain a cytotoxic CD8^+^ T cells response, as shown in Figure 2.

After a brief CTL response, CD8^+^ T cells die off and their tumor killing ability is lost until a new restimulation incident occurs. When CD4^+^ cells are stimulated and maintained, the CD8^+^ responses are sustained for long periods of time, improving the antitumor response, and potentially leading to the development of long-lasting immunological memory against tumor Ags. Recent studies have also shown the direct requirement for competent HLA class II pathway stimulation in the reduction of HLA class I-mediated response for an effective immunotherapy approach [18,70,71]. Furthermore, secondary stimulation following HLA class II-T cell receptor binding through costimulatory molecules is required for complete T cell stimulation. Strategies to boost co-stimulatory molecules in tumor vaccines would aid in T cell activation improving tumor reduction. One prostate cancer vaccine known as PROSTVAC (which consists of viral vectors expressing PSA) has recently been used to induce an immune response followed by a TRICOM vaccination for costimulatory signals that boost the antitumor immune response [42]. Clinical trials using PROSTVAC-TRICOM showed proof of principal with increased immune activation and a limited toxicity profile [42,43]. However, some issues arise from antivaccinia immune responses which could be counterproductive and may limit the use of viral vectors in vaccine design. Also, work performed in the laboratory of Susan Ostrand-Rosenberg has highlighted the importance of HLA class II vaccination strategies with increased expression of CD80 costimulatory molecules [17,72]. In their studies, tumor cells transduced with costimulatory molecules in the presence or absence of additional HLA class II molecules and the invariant chain (Ii), were capable of producing novel tumor-derived Ags and subsequent stimulation of CD4^+^ T cells [67]. Following the same vaccine design, GILT could also be transfected into HLA class II-positive prostate cancer cells with or without costimulatory molecules for the generation of tumor Ags due to GILT’s ability to increase acidic protease activity and Ag processing [37]. These studies also support the idea of modifying HLA class II pathway molecules as a means of generating novel pool of antigenic peptide repertoire. While prostate tumors express extremely low to undetectable levels of GILT, professional APCs express moderate to high levels of this protein in the host, which may alter HLA class II Ag presentation in the tumor microenvironment. The expression of GILT in the tumor microenvironment could also be altered upon chemotherapy or radiation therapy, and this should be analyzed carefully when designing immunotherapeutics. In any case, direct Ag presentation by tumor cells is very important and could be improved by upregulation of GILT and HLA class II proteins in the tumor as well as APCs in the tumor microenvironment. Alternatively, GILT DNA could be inserted in HLA class II positive prostate tumors and be tested as a whole-cell vaccine in boosting antitumor immune responses in the host.

## GILT’s Role in Ag Processing and T cell Recognition

Present throughout this review is the idea that tumor cells themselves add to the difficulty in developing effective immunotherapeutics. Their immune evasion mechanisms are so complex and diverse, and this review has only focused on remaking tumors as better targets for T cells. We have previously discussed how cysteinylation plays a role in immune evasion through oxidation reaction and differential display of peptides to T cells. Once this occurs, a reducing agent is needed to restore peptide functionality, one such reducing agent is GILT [73]. We and others have shown that GILT is an enzyme abundantly expressed by professional APC but is almost completely absent or expressed at very low levels in cancer cells [58,74]. The absence of GILT could result in incomplete processing of endogenous and exogenous Ags resulting in the display of a differential Ag repertoire on the surface of cancer cells [37,66]. Furthermore, the presence of GILT in tumor cells may enhance processing of cysteinylated peptides and aid in improved T cell activation and cancer cell recognition. GILT accomplishes this in the acidic endosomal and lysosomal compartments where HLA class II Ag processing and loading of peptide takes place [66,75]. In this compartment, GILT acts by enhancing and localizing the activity of cysteinyl and aspartyl cathepsins which are important proteases in peptide cleavage and folding [37,73]. Studies in melanoma have shown GILT’s ability to increase cathepsin activity and enhance Ag processing and presentation resulting in increased CD4^+^ T cell stimulation [37]. Though prostate tumors present more issues to immunotherapy than melanoma, preliminary studies in our laboratory have shown GILT insertion enhances HLA class II-restricted Ag presentation in prostate cancer cell lines (unpublished data). Given GILT’s role as a key element to Ag processing and epitope generation, if GILT can be effectively transfected into or upregulated by other means in prostate cancer cells then cancer cells could be restored back to their normal Ag presentation capability, increasing the effectiveness of prostate cancer immunotherapeutics and decreasing tumor immune evasion. Our laboratory is currently investigating this hypothesis and developing the strategies necessary for improved cancer immunotherapy. Although activation of the HLA class II pathway is important, the expression of immune-checkpoint proteins such as CTLA-4, PD-1 and PD-L1 [76,77] can be dysregulated by prostate tumors and escape immune recognition. Thus, inhibitors of these immune checkpoints specially monoclonal antibodies that block these proteins could be combined in designing prostate cancer immunotherapy. This review suggests that a combination therapy using stimulants of the HLA pathways and immune checkpoint blocker(s) could be useful in inducing strong antitumor immune responses in the host.

## Conclusions

In this review, we have discussed several immune evasion and restoration strategies that would definitely advance the field of prostate cancer immunobiology and immunotherapy. An outline for the selection of prostate tumor associated Ags is illustrated, highlighting the need for HLA class II-restricted tumor Ags inclusion in cancer vaccine studies. We have shown the importance of cysteinylation and its role in HLA class II-restricted epitope modification and Ag-induced T cell tolerance. The importance of the HLA class II pathway in designing novel immunotherapeutics has also been discussed and a case is made for its inclusion in future vaccine studies. GILT’s role in Ag processing and presentation has been reviewed to suggest how its manipulation could restore the Ag presenting characteristics of tumor cells. Inclusion of immune checkpoint blockers in designing immunotherapeutics is also discussed. Finally, these factors when taken together can be applied to improve the efficacy of immune responses against prostate cancer and may help shape new directions for future cancer vaccine development.

## Figures and Tables

**Table 1:** Prostate tumor associated antigens for immunotherapy.

Name	Functions
Prostate Specific Antigen (PSA)	Used as detection marker for prostate cancer. Tried in multiple immunotherapy vaccines. HLA class II epitopes have been identified in mouse models.
Prostatic Acid Phosphatase (PAP, PAcP)	Predates PSA as screening marker, particularly in cases of bone metastasis. HLA class II epitopes have been identified. Used as fusion protein with GM-CSF in Provenge DC therapy.
Prostate Specific Membrane Antigen (PSMA)	Membrane bound protein with increased expression in prostate cancer. Used as target for *in vivo* imaging and therapy techniques using monoclonal antibodies. HLA class II immunodominant epitope identified.
Telomerase (TERT, hTERT)	Increases telomere length leading to tumor progression and unchecked cell division. TERT protein has been identified as immunoreactive in TRAMP mouse model of prostate cancer. No HLA class II epitopes identified at this point.
Survivin (SVN, SUR)	Inhibitor of apoptosis in prostate cancer leading to increased cell division. HLA class II epitopes have been identified. Subject of multiple ongoing immunotherapy vaccine strategies.
Prostate Stem Cell Antigen (PSCA)	Cell-surface, glycosylphosphatidylinositol-anchored protein expressed in prostate cancer. PSCA is a target for peptide and antibody based therapy. No HLA class II epitopes identified at this point.

## References

[1] Carlsson S, Vickers AJ, Roobol M, Eastham J, Scardino P (2012). Prostate cancer screening: facts, statistics, and interpretation in response to the US Preventive Services Task Force Review. J Clin Oncol.

[2] Perez TY, Danzig MR, Ghandour RA, Badani KK, Benson MC (2015). Impact of the 2012 United States Preventive Services Task Force statement on prostate-specific antigen screening: analysis of urologic and primary care practices. Urology.

[3] Webster WS, Small EJ, Rini BI, Kwon ED (2005). Prostate cancer immunology: biology, therapeutics, and challenges. J Clin Oncol.

[4] Antonarakis ES, Armstrong AJ (2011). Emerging therapeutic approaches in the management of metastatic castration-resistant prostate cancer. Prostate Cancer Prostatic Dis.

[5] Mohebtash M, Madan RA, Gulley JL, Arlen PM (2008). Therapeutic prostate cancer vaccines: a review of the latest developments. Curr Opin Investig Drugs.

[6] Rozková D, Tiserová H, Fucíková J, Last'ovicka J, Podrazil M (2009). FOCUS on FOCIS: combined chemo-immunotherapy for the treatment of hormone-refractory metastatic prostate cancer. Clin Immunol.

[7] Cheever MA, Higano CS (2011). PROVENGE (Sipuleucel-T) in prostate cancer: the first FDA-approved therapeutic cancer vaccine. Clin Cancer Res.

[8] Brower V (2010). Approval of provenge seen as first step for cancer treatment vaccines. J Natl Cancer Inst.

[9] Anassi E, Ndefo UA (2011). Sipuleucel-T (provenge) injection: the first immunotherapy agent (vaccine) for hormone-refractory prostate cancer. P T.

[10] Slovin S (2015). Biomarkers for immunotherapy in genitourinary malignancies. Urol Oncol.

[11] Dickerson JB (2011). Provenge: revolutionary technology or ethical bust?. Hum Vaccin.

[12] Chambers JD, Neumann PJ (2011). Listening to Provenge--what a costly cancer treatment says about future Medicare policy. N Engl J Med.

[13] Baghdadi R (2010). Dendreon vs CMS: why the Provenge coverage controversy is bigger than just one product. Oncology (Williston Park).

[14] Carosella ED, Ploussard G, LeMaoult J, Desgrandchamps F (2015). A Systematic Review of Immunotherapy in Urologic Cancer: Evolving Roles for Targeting of CTLA-4, PD-1/PD-L1, and HLA-G. Eur Urol.

[15] Reese Z, Straubhar A, Pal SK, Agarwal N (2015). Ipilimumab in the treatment of prostate cancer. Future Oncol.

[16] Baxevanis CN, Papamichail M, Perez SA (2015). Prostate cancer vaccines: the long road to clinical application. Cancer Immunol Immunother.

[17] Thompson JA, Dissanayake SK, Ksander BR, Knutson KL, Disis ML (2006). Tumor cells transduced with the MHC class II Transactivator and CD80 activate tumor-specific CD4+ T cells whether or not they are silenced for invariant chain. Cancer Res.

[18] Thibodeau J, Bourgeois-Daigneault MC, Lapointe R (2012). Targeting the MHC Class II antigen presentation pathway in cancer immunotherapy. Oncoimmunology.

[19] Accolla RS, Tosi G (2012). Optimal MHC-II-restricted tumor antigen presentation to CD4+ T helper cells: the key issue for development of anti-tumor vaccines. J Transl Med.

[20] Knight D, Peterson AC, Rini BI, Harlin H, Gajewski TF (2009). The HLA-A2-restricted PSMA peptide LLHETDSAV is poorly immunogenic in patients with metastatic prostate cancer. Prostate.

[21] Basler M, Groettrup M (2007). Advances in prostate cancer immunotherapies. Drugs Aging.

[22] Klyushnenkova EN, Ponniah S, Rodriguez A, Kodak J, Mann DL (2004). CD4 and CD8 T-lymphocyte recognition of prostate specific antigen in granulomatous prostatitis. J Immunother.

[23] Saffran DC, Reiter RE, Jakobovits A, Witte ON (1999). Target antigens for prostate cancer immunotherapy. Cancer Metastasis Rev.

[24] Quintero IB, Araujo CL, Pulkka AE, Wirkkala RS, Herrala AM (2007). Prostatic acid phosphatase is not a prostate specific target. Cancer Res.

[25] Todorova K, Zoubak S, Mincheff M, Kyurkchiev S (2005). Biochemical nature and mapping of PSMA epitopes recognized by human antibodies induced after immunization with gene-based vaccines. Anticancer Res.

[26] Schroers R, Shen L, Rollins L, Xiao Z, Sonderstrup G (2003). Identification of MHC class II-restricted T-cell epitopes in prostate-specific membrane antigen. Clin Cancer Res.

[27] Wang XF, Kerzerho J, Adotevi O, Nuyttens H, Badoual C (2008). Comprehensive analysis of HLA-DR- and HLA-DP4-restricted CD4+ T cell response specific for the tumor-shared antigen survivin in healthy donors and cancer patients. J Immunol.

[28] Piesche M, Hildebrandt Y, Zettl F, Chapuy B, Schmitz M (2007). Identification of a promiscuous HLA DR-restricted T-cell epitope derived from the inhibitor of apoptosis protein survivin. Hum Immunol.

[29] Friedrichs B, Siegel S, Andersen MH, Schmitz N, Zeis M (2006). Survivin-derived peptide epitopes and their role for induction of antitumor immunity in hematological malignancies. Leuk Lymphoma.

[30] Filaci G, Fravega M, Setti M, Traverso P, Millo E (2006). Frequency of telomerase-specific CD8+ T lymphocytes in patients with cancer. Blood.

[31] McNeel DG, Nguyen LD, Disis ML (2001). Identification of T helper epitopes from prostatic acid phosphatase. Cancer Res.

[32] Haque MA, Li P, Jackson SK, Zarour HM, Hawes JW (2002). Absence of gamma-interferon-inducible lysosomal thiol reductase in melanomas disrupts T cell recognition of select immunodominant epitopes. J Exp Med.

[33] Khong HT, Wang QJ, Rosenberg SA (2004). Identification of multiple antigens recognized by tumor-infiltrating lymphocytes from a single patient: tumor escape by antigen loss and loss of MHC expression. J Immunother.

[34] Amria S, Hajiaghamohseni LM, Harbeson C, Zhao D, Goldstein O (2008). HLA-DM negatively regulates HLA-DR4-restricted collagen pathogenic peptide presentation and T cell recognition. Eur J Immunol.

[35] Dolan BP, Gibbs KD, Ostrand-Rosenberg S (2006). Tumor-Specific CD4+ T cells Are Activated by "Cross-Dressed" Dendritic Cells Presenting Peptide-MHC CLass II Complexes Acquired from Cell-Based Cancer Vaccines. J Immunol.

[36] Younger AR, Amria S, Jeffrey WA, Mahdy AE, Goldstein OG (2008). HLA class II antigen presentation by prostate cancer cells. Prostate Cancer Prostatic Dis.

[37] Goldstein OG, Hajiaghamohseni LM, Amria S, Sundaram K, Reddy SV (2008). Gamma-IFN-inducible-lysosomal thiol reductase modulates acidic proteases and HLA class II antigen processing in melanoma. Cancer Immunol Immunother.

[38] Mennuni C, Ugel S, Mori F, Cipriani B, Iezzi M (2008). Preventive vaccination with telomerase controls tumor growth in genetically engineered and carcinogen-induced mouse models of cancer. Cancer Res.

[39] Klyushnenkova EN, Kouiavskaia DV, Kodak JA, Vandenbark AA, Alexander RB (2007). Identification of HLA-DRB1*1501-restricted T-cell epitopes from human prostatic acid phosphatase. Prostate.

[40] Chavan PR, Chavan SV, Chavan NR, Trivedi VD (2009). Detection rate of prostate cancer using prostate specific antigen in patients presenting with lower urinary tract symptoms: a retrospective study. J Postgrad Med.

[41] Thompson IM, Tangen CM, Kristal AR (2008). Prostate-specific antigen: a misused and maligned prostate cancer biomarker. J Natl Cancer Inst.

[42] Harzstark AL, Small EJ (2009). Immunotherapeutics in development for prostate cancer. Oncologist.

[43] Sobol I, Thompson RH, Dong H, Krco C, Kwon ED (2015). Immunotherapy in prostate cancer. Curr Urol Rep.

[44] Machlenkin A, Paz A, Bar Haim E, Goldberger O, Finkel E (2005). Human CTL epitopes prostatic acid phosphatase-3 and six-transmembrane epithelial antigen of prostate-3 as candidates for prostate cancer immunotherapy. Cancer Res.

[45] Fang LC, Dattoli M, Taira A, True L, Sorace R (2008). Prostatic acid phosphatase adversely affects cause-specific survival in patients with intermediate to high-risk prostate cancer treated with brachytherapy. Urology.

[46] Dannull J, Diener PA, Prikler L, Fürstenberger G, Cerny T (2000). Prostate stem cell antigen is a promising candidate for immunotherapy of advanced prostate cancer. Cancer Res.

[47] Gu Z, Thomas G, Yamashiro J, Shintaku IP, Dorey F (2000). Prostate stem cell antigen (PSCA) expression increases with high gleason score, advanced stage and bone metastasis in prostate cancer. Oncogene.

[48] Taeb J, Asgari M, Abolhasani M, Farajollahi MM, Madjd Z (2014). Expression of prostate stem cell antigen (PSCA) in prostate cancer: a tissue microarray study of Iranian patients. Pathol Res Pract.

[49] Olafsen T, Gu Z, Sherman MA, Leyton JV, Witkosky ME (2007). Targeting, imaging, and therapy using a humanized antiprostate stem cell antigen (PSCA) antibody. J Immunother.

[50] Matsueda S, Kobayashi K, Nonaka Y, Noguchi M, Itoh K (2004). Identification of new prostate stem cell antigen-derived peptides immunogenic in HLA-A2(+) patients with hormone-refractory prostate cancer. Cancer Immunol Immunother.

[51] Brower V (2010). Telomerase-based therapies emerging slowly. J Natl Cancer Inst.

[52] Bachinsky MM, Guillen DE, Patel SR, Singleton J, Chen C (2005). Mapping and binding analysis of peptides derived from the tumor-associated antigen survivin for eight HLA alleles. Cancer Immun.

[53] Casati C, Dalerba P, Rivoltini L, Gallino G, Deho P (2003). The apoptosis inhibitor protein survivin induces tumor-specific CD8+ and CD4+ T cells in colorectal cancer patients. Cancer Res.

[54] Pisarev V, Yu B, Salup R, Sherman S, Altieri DC (2003). Full-length dominant-negative survivin for cancer immunotherapy. Clin Cancer Res.

[55] Kim EK, Cho HI, Yoon SH, Park MJ, Sohn HJ (2008). Efficient generation of survivin-specific cytotoxic T lymphocytes from healthy persons in vitro: quantitative and qualitative effects of CD4+ T cells. Vaccine.

[56] Widenmeyer M, Griesemann H, Stevanović S, Feyerabend S, Klein R (2012). Promiscuous survivin peptide induces robust CD4+ T-cell responses in the majority of vaccinated cancer patients. Int J Cancer.

[57] Wang XF, Kerzerho J, Adotevi O, Nuyttens H, Badoual C (2008). Comprehensive analysis of HLA-DR- and HLA-DP4-restricted CD4+ T cell response specific for the tumor-shared antigen survivin in healthy donors and cancer patients. J Immunol.

[58] Phan UT, Arunachalam B, Cresswell P (2000). Gamma-interferon-inducible lysosomal thiol reductase (GILT). Maturation, activity, and mechanism of action. J Biol Chem.

[59] Haque MA, Hawes JW, Blum JS (2001). Cysteinylation of MHC class II ligands: peptide endocytosis and reduction within APC influences T cell recognition. J Immunol.

[60] Amria S, Cameron C, Stuart R, Haque A (2008). Defects in HLA class II antigen presentation in B-cell lymphomas. Leuk Lymphoma.

[61] Haque A, Blum JS (2005). New insights in antigen processing and epitope selection: development of novel immunotherapeutic strategies for cancer, autoimmunity and infectious diseases. J Biol Regul Homeost Agents.

[62] Gerritsen WR (2012). The evolving role of immunotherapy in prostate cancer. Ann Oncol.

[63] Nanda NK, Birch L, Greenberg NM, Prins GS (2006). MHC class I and class II molecules are expressed in both human and mouse prostate tumor microenvironment. Prostate.

[64] Mishra DK, Chen Z, Wu Y, Sarkissyan M, Koeffler HP (2010). Global methylation pattern of genes in androgen-sensitive and androgen-independent prostate cancer cells. Mol Cancer Ther.

[65] God JM, Zhao D, Cameron CA, Amria S, Bethard JR (2014). Disruption of HLA class II antigen presentation in Burkitt lymphoma: implication of a 47,000 MW acid labile protein in CD4+ T-cell recognition. Immunology.

[66] God JM, Cameron C, Figueroa J, Amria S, Hossain A (2015). Elevation of c-MYC disrupts HLA class II-mediated immune recognition of human B cell tumors. J Immunol.

[67] Thompson JA, Srivastava MK, Bosch JJ, Clements VK, Ksander BR (2008). The absence of invariant chain in MHC II cancer vaccines enhances the activation of tumor-reactive type 1 CD4+ T lymphocytes. Cancer Immunol Immunother.

[68] Dow C, Oseroff C, Peters B, Nance-Sotelo C, Sidney J (2008). Lymphocytic choriomeningitis virus infection yields overlapping CD4+ and CD8+ T-cell responses. J Virol.

[69] Knutson KL, Disis ML (2005). Tumor antigen-specific T helper cells in cancer immunity and immunotherapy. Cancer Immunol Immunother.

[70] Neeley YC, McDonagh KT, Overwijk WW, Restifo NP, Sanda MG (2002). Antigen-specific tumor vaccine efficacy in vivo against prostate cancer with low class I MHC requires competent class II MHC. Prostate.

[71] Klyushnenkova EN, Kouiavskaia DV, Berard CA, Alexander RB (2009). Cutting edge: permissive MHC class II allele changes the pattern of antitumor immune response resulting in failure of tumor rejection. J Immunol.

[72] Bosch JJ, Thompson JA, Srivastava MK, Iheagwara UK, Murray TG (2007). MHC class II-transduced tumor cells originating in the immune-privileged eye prime and boost CD4(+) T lymphocytes that cross-react with primary and metastatic uveal melanoma cells. Cancer Res.

[73] Arunachalam B, Phan UT, Geuze HJ, Cresswell P (2000). Enzymatic reduction of disulfide bonds in lysosomes: characterization of a gamma-interferon-inducible lysosomal thiol reductase (GILT). Proc Natl Acad Sci U S A.

[74] Haque MA, Li P, Jackson SK, Zarour HM, Hawes JW (2002). Absence of gamma-interferon-inducible lysosomal thiol reductase in melanomas disrupts T cell recognition of select immunodominant epitopes. J Exp Med.

[75] O'Donnell PW, Haque A, Klemsz MJ, Kaplan MH, Blum JS (2004). Cutting edge: induction of the antigen-processing enzyme IFN-gamma-inducible lysosomal thiol reductase in melanoma cells Is STAT1-dependent but CIITA-independent. J Immunol.

[76] Iwai Y, Ishida M, Tanaka Y, Okazaki T, Honjo T (2002). Involvement of PD-L1 on tumor cells in the escape from host immune system and tumor immunotherapy by PD-L1 blockade. Proc Natl Acad Sci U S A.

[77] Dolan DE, Gupta S (2014). PD-1 pathway inhibitors: changing the landscape of cancer immunotherapy. Cancer Control.

